# 3D margin assessment predicts local tumor progression after ablation of colorectal cancer liver metastases

**DOI:** 10.1080/02656736.2022.2055795

**Published:** 2022

**Authors:** Nikiforos Vasiniotis Kamarinos, Mithat Gonen, Vlasios Sotirchos, Elena Kaye, Elena N. Petre, Stephen B. Solomon, Joseph P. Erinjeri, Etay Ziv, Assen Kirov, Constantinos T. Sofocleous

**Affiliations:** a Department of Interventional Oncology, Memorial Sloan Kettering Cancer Center, New York, NY, USA; b Department of Epidemiology and Biostatistics, Memorial Sloan Kettering Cancer Center, New York, NY, USA; c Department of Medical Physics, Memorial Sloan Kettering Cancer Center, New York, NY, USA

**Keywords:** Thermal ablation, ablation margin assessment, colorectal cancer liver metastases

## Abstract

**Objective:**

To determine the feasibility and prognostic value of 3D measuring of the ablation margins using a dedicated image registration software.

**Methods:**

This retrospective study included 104 colorectal liver metastases in 68 consecutive patients that underwent microwave ablation between 08/2012 and 08/2019. The minimal ablation margin (MM) was measured in 2D using anatomic landmarks on contrast enhanced CT(CECT) 4–8 weeks postablation, and in 3D using an image registration software and immediate post-ablation CECT. Local tumor progression (LTP) was assessed by imaging up to 24 months after ablation. A blinded interventional radiologist provided feedback on the possibility of additional ablation after examining the 3D-margin measurements.

**Results:**

The 3D-margin assessment was completed in 79/104 (76%) tumors without the need for target manipulation. In 25/104 (24%) tumors, manipulation was required due to image misregistration. LTP was observed in 40/104 (38.5%) tumors: 92.5% vs 7.5% for those with margin <5mm vs ≥5mm, respectively (*p*=0.0001). The 2D and 3D-assessments identified margin <5mm in 17/104 (16%), and in 74/104 (71%) ablated tumors, respectively (*p* < 0.01). The sensitivity and specificity of the 3D software for predicting LTP was 93% (37/40) and 42% (27/64), respectively. Additional ablation to achieve a MM of 5mm would have been offered in 26/37 cases if the 3D-margin assessment was available intraoperatively.

**Conclusion:**

Image registration software can measure ablation margins and detect MM under 5mm intraoperatively, with significantly higher sensitivity than the 2D technique using landmarks on the post-ablation CECT. The identification of a margin under 5mm is strongly associated with LTP.

## Introduction

Percutaneous image-guided thermal ablation (TA) is a safe and effective local treatment for selected patients with colorectal liver metastases (CLM) [1–4]. Despite the favorable safety profile and curative potential, the historically high local tumor progression (LTP) rates ranging between 2.8 and 60% remain the main limitation to the widespread use of ablation as a local cure for CLM [3].

The ability of ablation to eradicate the tumor with sufficient margins is probably the most important technical factor for optimal outcomes [2,5–9]. The minimal ablation margin (MM) is defined as the shortest distance between the tumor and the ablation zone border [10]. Specifically, it has been shown that a 5mm MM all around the target CLM is critical to achieving local tumor control [2,11,12].

Accurate intraprocedural margin assessment provides valuable feedback to the operator at the time of treatment [13]. Ablation margins have been historically measured manually in 2D by estimating the distance between the radiographic boundaries of the tumor and the ablation zone in the 4–8 weeks post-ablation contrast-enhanced scan using anatomic landmarks [10]. Although this method accounts for the resolution of immediate post-ablation change, it can be complicated by positional differences in the scans performed before and after ablation, at different time points. In addition, it is time-consuming and thus unlikely to be used intraoperatively, precluding real-time feedback for additional ablation at the time of treatment [13].

The measurement of ablation margins in 3D is an accurate and reliable method of margin assessment [13–18]. Precisely, multiplanar, stereotactic volumetric models had a significantly improved discrimination power detecting insufficient ablation margins and, consequently, LTP [13,14]. The goal of this study is to determine the feasibility and prognostic value of measuring ablation margins in 3D using pre and postablation scans performed on the day of the procedure. The specific ablation confirmation software used in this study is evaluated as a potential intraoperative tool of assessment of the ablation zone (AZ) and margins, that could help predict subsequent LTP after tumor ablation.

## Methods

### Study population

This retrospective study was approved by the institutional review board, and informed consent was waived. It included 79 consecutive patients with 122 colorectal liver metastases treated with thermal ablation between August 2012 and August 2019. All patients had up to three CLM (each < 5 cm in largest diameter) and no more than three extrahepatic sites of disease (including lymph nodes and pulmonary nodules). Ablation was offered according to institutional and Interventional Radiology standards of practice. The ablation plan was based on tumor characteristics, anatomical considerations, and the general condition of the respective patient. CLM diagnosis was confirmed by imaging on multiphasic contrast MRI or CT, PET/CT, and histopathological examination before or during the ablation procedure. In our institution, patients that cannot undergo general anesthesia and those with uncorrectable coagulopathy (international normalized ratio [INR] > 1.5 or platelet count <50,000/mm3) are not eligible to be treated with thermal ablation.

### Ablation procedure and follow-up

All procedures were performed under general anesthesia with continuous hemodynamic monitoring by an anesthesiologist. In all cases, the manufacturer-recommended ablation protocol for the desired ablation zone size was applied and completed. At the minimum, a non-contrast-enhanced CT was performed immediately before, and a triphasic CT scan was performed immediately after the ablation for all tumors [19]. For both intraprocedural scans, a 2.5–3mm slice thickness was used. Applicator placement and tumor targeting were conducted under CT guidance. Additional CT-fluoroscopy, real-time PET/CT and ultrasound guidance were available and used as needed [3,20]. Overlapping ablations were performed to create an ablation zone (AZ) with a minimum ablation margin of ≥5mm all around the target tumor [19]. All patients underwent a triphasic CT performed within 4–8 weeks after the ablation and were followed up with subsequent scans at 2–4-month intervals for up to 2 years [19]. Evidence of tumor within 1 cm from the AZ seen on contrast-enhanced CT, PET/CT, and/or contrast MRI was considered LTP [19].

Specific exclusion criteria were utilized to determine the final cohort in this study, including:

1. Thermal ablation modalities other than microwave ablation (i.e., radiofrequency ablation)
2. Unavailability of the minimum CT scan requirements described above (intraprocedural and follow-up scans), and
3. CT image slice thickness greater than 3 mm.

### 3D margin assessment as a predictor of LTP

The 3D margin assessment was performed with the scientific research version of Ablation Confirmation (NeuWave Medical, Madison, WI) software, which uses an intensity-based deformation algorithm. Specifically, it attempts to match areas with similar gradient changes. The most significant gradient changes are at the edges of structures such as organ boundaries. After registration, the tumor and the AZ were always segmented in the pre, and post-ablation CT scan respectively. Both scans were always performed during the procedure and were similar in terms of patient and operating table position, slice thickness size, and the number of image slices. After reconstruction in all anatomical planes, segmentations were performed with the automatic and/or semiautomatic selection feature of the software that allows selection editing. When the tumor was not visible in the non-contrast-enhanced pre-ablation CT scan, semi-automatic segmentation was employed by editing the initial auto-segmentation in all three planes using the most recent available imaging scan (always available within 30 days from ablation) in which the tumor was visible, as guidance. The post-ablation scan was contrast-enhanced in all cases and was always performed at the end of the ablation.

**Figure 1.**
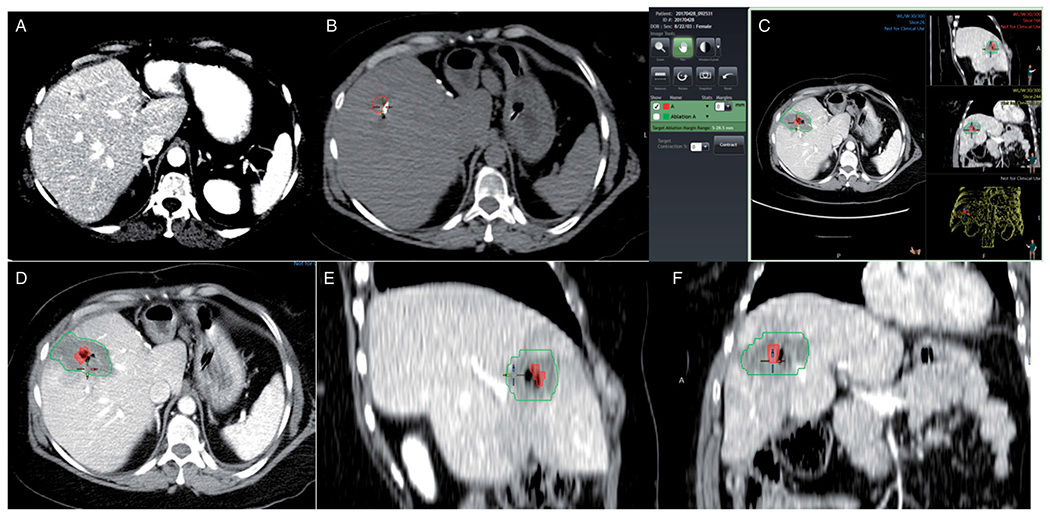
Measurement of ablation margins in 3 D in a patient with colorectal liver metastasis in segment 8. Pre-ablation contrast-enhanced CT scan 2 days before the procedure (A). Segmentation of the tumor (red) in the pre-ablation CT scan of the day of the procedure (B). General view of the software interface for ablation margin assessment (C). Detail of the tumor (red) and the ablation zone (green) in axial, sagittal, and coronal plane (D–F), showing adequate ablation margins in all three planes.

The minimal size of the ablation margin was calculated automatically in millimeters after non-rigid registration of the pre-and post-ablation images without applying contraction Figure 1. All 3D minimal margin measurements were repeated using the embedded tissue contraction algorithm, with a uniform contraction factor of 15%, to account for immediate post-ablation tissue contraction.

**Figure 2.**
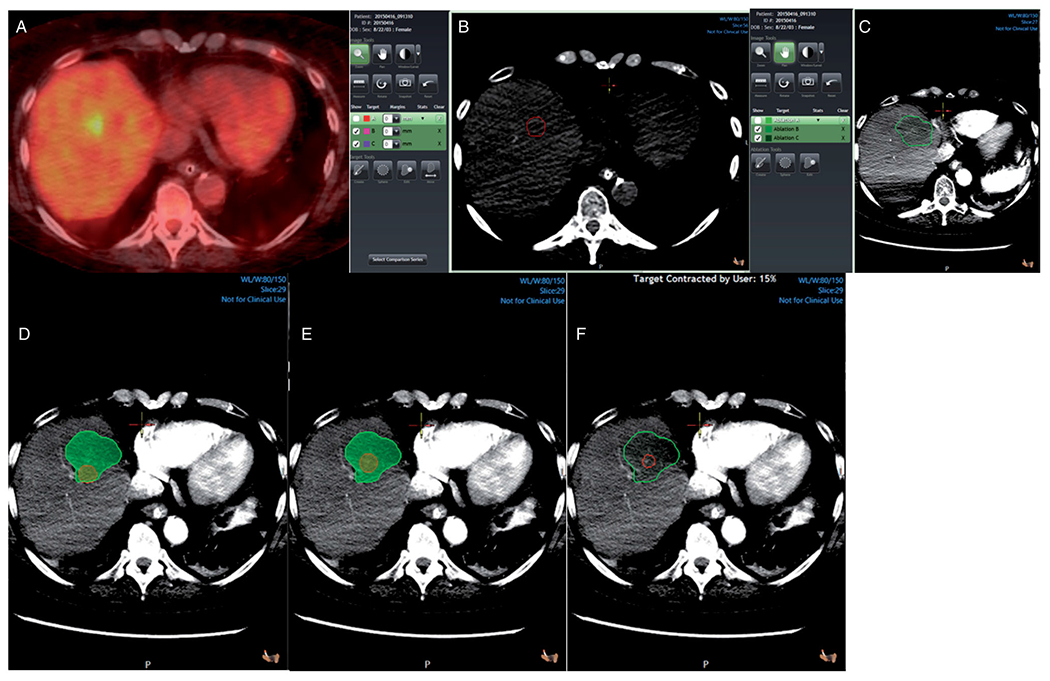
3 D margin assessment in a patient with colorectal liver metastasis in segment 8. Pre-ablation PET/CT scan showing the tumor (A). Tumor segmentation (red) in a pre-ablation CT scan (B). Ablation zone segmentation (green) in a post-ablation contrast-enhanced CT scan (C). Tumor (red) and ablation zone (green) registration without target movement showing ablation margins <5mm (D). Tumor (red) and ablation zone (green) registration with target movement to match anatomic landmarks (E). Tumor (red) and ablation zone (green) registration with target movement and tissue contraction algorithm applied (F).

The software provides a visual indication of the registration accuracy with a pseudo-colored overlay of the two scans (pre and post-ablation) that show areas of good alignment vs. misalignment to guide corrections when and as needed. When image registration could not achieve acceptable alignment, the segmented tumor was moved to match the corresponding anatomic landmarks Figure 2. Landmarks used included intrahepatic vessel bifurcations, small benign lesions (i.e., calcifications, cysts), indentations or bulge points of the liver capsule, and surgical staples, as previously described [10]. For subcapsular tumors, the minimal margin was measured manually in 3D, instead of automatically, after registering the segmented tumor and the AZ and accounting for the location of the liver capsule. The entire process from segmentation to minimal margin calculation with or without application of tissue contraction algorithm was recorded.

To further evaluate the 3D software effectiveness as an intraprocedural tool, a blinded faculty interventional radiologist with over 20 years of experience in tumor ablation, examined all minimal margin measurements <5mm and provided feedback on whether additional ablation could be offered. This process was completed using the 3D tumor and ablation zone segmentations without applying a tissue contraction algorithm.

The primary endpoint of the study was to evaluate the diagnostic accuracy of the intraprocedural 3D ablation margin measurements as a predictor of LTP at 2 years. As a secondary endpoint, all 3D measurements were compared to minimal margin measurements made with the conventional 2D method. Manual 2D margin assessment was carried out by comparing the diagnostic contrast-enhanced pre-ablation CT images of the tumor performed within 30 days before the ablation, and the post-ablation contrast-enhanced CT images of the ablation zone obtained 4–8 weeks after the ablation, as previously described and as recommended in guidelines for reporting standards for tumor ablation [10,19]. Although the timing of the images used in the 3D and the 2D assessments were different (intraprocedural versus 4–8 weeks, respectively), this was specifically designed to evaluate whether the immediate assessment can accurately identify the margin and predict local tumor progression at least as effective as the previously described 2D assessment performed with the use of the first post-ablation CECT 4–8 weeks later despite the challenges of the immediate post-ablation changes (e.g., hyperemia, bleeding, organ mobilization or deformity).

### Statistical analysis

Minimal margin measurements were categorized as ≥5mm or <5 mm. Detection rates of a minimal margin <5mm were calculated for both 3D and 2D assessment methods. As most LTP events are known to occur within two years after ablation, the diagnostic accuracy of each method to predict the 2-year LTP rate was calculated [2]. Sensitivity, specificity, and accuracy were measured separately for each margin assessment method and compared using the chi-square test.

## Results

After applying the exclusion criteria described above, 104/122 CLM in 68/79 patients were eligible for the 3D assessment evaluation (Table 1). A 3D margin assessment was completed in 79/104 (76%) tumors without target movement, and in 25/104 (24%) tumors with target manipulation since image registration did not achieve acceptable anatomic alignment.

The 3D method identified a margin size under 5mm in 34/104 and 74/104 ablated tumors with and without tissue contraction algorithm application respectively. The 2D and 3D (without contraction) assessments identified minimal margin (MM) size under 5mm in 17/104 (16%) and in 74/104 (71%) ablated tumors, respectively (*p*<0.01). The entire process from segmentation to minimal margin calculation, with or without application of tissue contraction algorithm, took an average time of 10 (range 5–15) minutes depending on the required manual correction.

**Figure 3.**
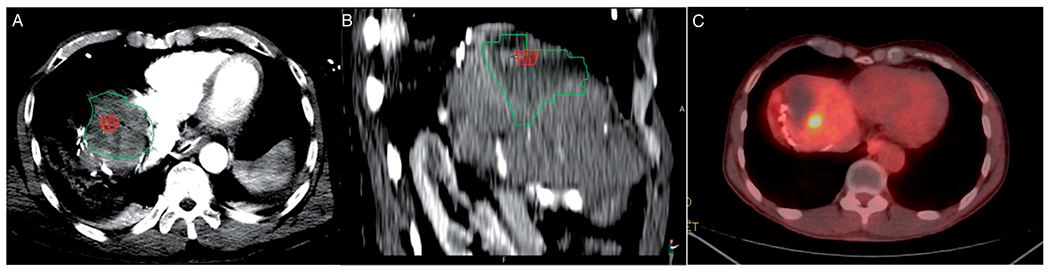
Colorectal metastasis (red) ablation in the liver dome. Ablation zone (green) with adequate margins in the axial plane (A), but suboptimal margins in sagittal plane examination (B). Local tumor progression is evident on a 10-month PET/CT follow-up scan (C).

The median follow-up time was 21 (range: 1, 24) months. Local tumor progression (LTP) was observed in 40/104 (38.5%) tumors; with LTP of 92.5% vs 7.5% corresponding to minimal margin <5mm vs >5mm (*p*=0.0001) respectively; within the 24-month follow-up period of the study Table 2 and Figure 3.

The sensitivity, specificity, and accuracy of the 3D software for predicting LTP was 50% (20/40), 78% (50/64) and 67% (70/104), or 93% (37/40), 42% (27/64), and 62% (64/104), with or without tissue contraction, respectively. The 2D method had a sensitivity and specificity of 20% (8/40) and 86% (55/64), respectively. The accuracy of the 2D method was 61% (63/104). Sensitivity and specificity were 96% (25/26) and 32% (17/53) in the group of tumors where the registration was performed solely by the software, without any manual alignment and without contraction.

The difference of the 3D method without contraction vs. the 2D method in terms of sensitivity (*p*<.001), and specificity (*p*<.001) was statistically significant. Additional ablation would be offered in 26/37 cases with a minimal margin <5mm and LTP if the software was available on the day of the procedure. Tumor proximity to critical vessels or central bile ducts precluded further ablation for the remaining 11 tumors.

## Discussion

This study describes the evaluation of ablation margins in 3D with image registration software using intraprocedural CT scans. The workflow is simple and consists of 3D registration and segmentation of the tumor and ablation zone (AZ) and automated computation of the minimal margin (MM).

Several studies have analyzed the risk factors and patterns of LTP in an effort to improve oncologic outcomes after thermal ablation (TA) [2,5,6,8–10]. Similar to the surgical margin, the ablation margin is critical for local tumor control [2,5,9,10,12,21]. Unlike resected specimens, the evaluation of the AZ and margin depend on imaging. The MM represents the thickness of normal tissue interposed between the tumor edge and the AZ and is traditionally measured in 2D [10]. Local progression of tumors with adequate 2D margins led many investigators to study the feasibility of accurate margin measurement in 3D with the registration of pre and postablation images [13,15,17,18]. All studies have shown that the 3D assessment identified more ablations with no margins or margins less than 5mm compared to the conventional 2D method [13,15,17,18].

The present study shows improved sensitivity in suboptimal margin detection and prediction of LTP with the 3D AC software using the intraprocedural scans compared to the conventional 2D manual method (2D). The comparison with the 2D performed on the first post-ablation scan 4–8 weeks later was intentional. This decision intended to show that the intraprocedural 3D assessment would be at least as accurate as of the later 2D assessment that has been used by us and others for years and is compliant with 2014 guidelines for reporting standards for ablation [19]. The 2014 recommendations require at least 3 weeks to elapse from the ablation in order to allow accurate ablation zone and margin calculation and determine ablation efficacy, after the resolution of known post-ablation changes such as hyperemia, inflammation and occasional bleeding. Moreover, both for RF and microwave ablation and both for cirrhotic and non-cirrhotic liver, the ablation zone shows a rapid decrease in size of around 30% at one month [22]. As such, the immediate assessment could arguably be less accurate than the later 2D assessment. The current study indicates that, at least in terms of margin calculation and LTP prediction, the intraoperative AC software assessment was more sensitive than the 2D evaluation using the recommended first postablation CECT.

To account for tissue contraction, the software can calculate ablation margins by applying a 1–30% contraction factor. The contraction algorithm applies contraction to the entire volume of the tumor. This feature automatically reduces the dimensions of the segmented tissue uniformly to simulate the immediate post-ablation tissue shrinkage. The decision to use or not to use contraction and the percentage of contraction to be applied is made by the physician/operator in each case based on their best clinical judgment. To our knowledge, although multiple studies are describing the effects of these factors on post-ablation tissue shrinkage, there is currently no standardized method available to calculate tissue contraction. In this study, in the analysis of 3D measurements with contraction, a contraction factor of 15% was used uniformly in all cases to assess the software’s contraction feature performance. The sensitivity of the 3D method in detecting minimal margins <5mm and subsequently LTP was higher when the tissue contraction algorithm was not applied. In addition, the intraoperative 3D method without contraction was more sensitive than the 2D method for detection of margins <5mm (*p*<.001), although the 2D method was more specific (*p*<.001). This could be explained by the partial involution and healing of the AZ by the time the 2D assessment post-ablation scan is performed [19].

The estimated margin increased when the contraction algorithm was enabled. Therefore, some of the false positives became true negative, and overall, the numbers of true positives and false-positive decreased and the numbers of true negative and false negatives increased. The tissue contraction feature improved the overall accuracy but reduced the sensitivity of the 3D measurements from 93% to 50%. As such, cases that would benefit from additional ablation were evaluated without using the tissue contraction algorithm. Notably, in 26/37 cases, additional ablation would have been offered to achieve adequate margins. Generally, in the case of suboptimal margins, it is preferable to safely extend the AZ in the same sitting to avoid a repeat ablation in the future or progression in a pattern that makes ablation difficult or impossible. Additional ablation is not performed when extending the AZ is risky or not feasible.

The 3D software simplifies the margin assessment workflow by allowing automatic (and/or semi-automatic) segmentation and measurements. Compared to the conventional 2D method, the 3D evaluation is much faster and helps visualize the contour and the volume of both the target tumor and the respective AZ in images acquired with the same parameters on the day of the ablation [10].

A critical technical consideration of image registration software is that pre-and post-ablation imaging studies should be similar in slice thickness, image number, patient and table position to decrease registration error. This is not always possible, especially in cases with hydro- or pneumo-dissection and organ or table movement. In these cases, the segmented tumor needs to be moved to preserve the relative distances to other anatomical structures [10]. The subcapsular location of hepatic tumors can also increase the inaccuracy of margin measurements as image registration software cannot discriminate subcapsular from intra-parenchymal tumors. Ablation margins in tumors located <5mm from the liver capsule were measured with the software manually in all three anatomical planes, accounting for the location of the liver capsule. A more sophisticated, automated measurement method that involves segmentation of the liver capsule has been proposed and is associated with promising outcomes [15,23].

Studies using state-of-the-art image registration software to assess ablation margins in 3D showed similar findings [13,16,24]. Solbiati et al. registered pre-ablation and 24 h post-ablation CT scan, and classified HCC ablation 3D measurements in four volumetric categories: 100%, 90–99%, 50–89%, or 0–49% of the intended 5mm ablative margin. The authors reported a 100% LTPFS at one year for patients with a 100% 5mm ablation margin for HCC [24]. Using the same registration software and similar methodology, Laimer et al. correlated 3D margin measurements of 76 ablated CLM in 45 patients with LTP [16]. Precisely, their findings showed no LTP for ablated tumors with a 5mm margin all-around 95% of tumor volume. The authors also concluded that local control could be achieved with a 3mm margin all around the entire (100%) tumor volume.

Although volumetric margin classifications less than 100% can be justified and is accepted for the area of (subcapsular) tumors near the capsule or close to large central vessels (perivascular tumors) and bile ducts or other critical anatomic structures, an ablated tumor with a 5mm margin in less than its entire volume translates to a 2D AZ with a MM <5mm and thus at high risk for LTP [2,8,10]. On the other hand, the size of the ablation volumes calculated by the 3D software may be underestimated due to tissue shrinkage, especially after microwave ablation, a fact that can explain the absence of local progression for <5mm margins or >5mm margins in <100% of tumor volume [16,24].

Another study that measured ablation margins in 3D and performed a volumetric analysis separately confirmed that the 3D method had higher discrimination power for detecting 2-year LTP compared to the 2D method [13]. The authors also compared 3D MM measurements to volumetric measurements of insufficient coverage corresponding to 5mm ablation margins and found no statistically significant difference in the discrimination power of both methods (*p*=0.06). They also pointed out that large ablation zone volumes without adequate minimum margins exhibited LTP. This last observation indicates the importance of not only a sufficient but well-centered/targeted AZ around the target tumor.

Both 2D and 3D MM and volumetric measurements rely on imaging, and the critical 1–2mm distance that can turn adequate ablation margins to suboptimal can easily be miscalculated. In addition, ROC analysis of 3D measurements showed poor accuracy in predicting LTP for suboptimal margins of 1–2mm [16]. The combination of margin measurement with a post-ablation pathological examination of the AZ is the most objective method to establish a complete tumor ablation with local curative intent [25,26]. Again, 3D software can help visualize the tumor and the AZ after segmentation and image registration and guide post-ablation biopsies to the minimal margin. The software used in this study offers the possibility of additional registration of ablation applicators and biopsy needles to target the tumor, the center and MM of the AZ. This feature was not used in this retrospective study, but it offers a valuable tool for prospective projects that could assess not only the margin but complete tumor necrosis or residual viable tumor within the ablation zone [25–28].

This study demonstrated that the 3D assessment of ablation margins using intraprocedural scans compare favorably with the standard-of-care 2D manual examination of margins which is performed using 3–8-week post-ablation scans, as suggested by the ablation guidelines for reporting standards [19]. Limitations of this study lie in its retrospective design. Other available intraprocedural imaging studies such as PET/CT, CT Fluoro, and the US could not be used even if available, since the software version used allows segmentation and registration of CT studies only. Contrast-enhanced intraprocedural pre-ablation scans were not available in this retrospective cohort; contrast-enhancement of the target tumor could have made the automatic registration feature of the software easier. In cases where the tumor was not conspicuous enough in the immediate pre-ablation scan, the automatic registration was corrected by using a contrast-enhanced pre-ablation scan performed within 30 days before the ablation as guidance. While we acknowledge this as a limitation for evaluating the software’s capabilities, we believe that the registration should always be examined in all anatomical planes and corrected when needed. Generally, registration has limitations with all available software platforms and the software used in this study is no exception. However, the ability to register using reliable anatomic landmarks is an important feature and can be used to improve registration with this software. Reliable anatomic landmarks for registration have been described in prior work [10] and could help improve registration even when using 3D software platforms similar to the one presented here. The software methodology allows the operator to optimize registration by applying these landmarks, and this step can be a part of the registration process. To account for the limitation of this subjective input we have analyzed the sensitivity and specificity after excluding the cases where registration was improved by manual registration. The results (96% and 32% for sensitivity and specificity, respectively) lay within the 95% confidence interval of the sensitivity and specificity of the entire sample. Finally, the number of antennas, the power and the duration of ablation, the use of hydrodissection, a subcapsular tumor location, and minimal breathing movements are some of the factors that can cause anatomical structure movement and, thus, registration error. The number of events in this retrospective cohort was small and did not allow correlations with those factors. Future prospective studies are needed to confirm the findings and address the related limitations.

In conclusion, this study confirms the feasibility of 3D assessment of the AZ using imaging scans performed immediately before and after the ablation. The 3D measurement of the MM is more accurate than the conventional 2D method and highly sensitive to detecting suboptimal margins and LTP. The image registration software can be used intraprocedural and guide decisions about additional ablation.

## Figures and Tables

**Table 1.** Patient and tumor characteristics.

Patient (*n*=68) and Tumor (*n*104) characteristics	
Characteristic	Value
Age (y)*	57.5 (32–78)
Gender	
Female	23 (34)
Male	45 (66)
Race	
Asian/Far East/Indian Subcontinent	3 (4)
Black/African American	4 (5)
Patient refused to answer	2 (3)
White	59 (88)
Tumor size (cm)**	1.9 (0.8–4.0)
Time between diagnosis of colorectal cancer and ablation (mo)*	31.5 (8–163)
No. of tumors treated per patient**	1.5 (1–5)

Unless otherwise indicated, data represent the number of patients, and data in parentheses are percentages.

* Data are median values, and data in parentheses represent the range.

** Data are mean values, and data in parentheses represent the range.

**Table 2.** Performance of ablation margin assessment methods.

	LTP (total 40)	No LTP (total 64)	Total number of tumors	Sensitivity	Specificity	Accuracy
Minimal margin <5mm – 3 D with tissue contraction	20	14	34	50% (95% CI: 35,65)	78% (95% CI: 68,88)	67% (95% CI: 58,76)
Minimal margin ≥5mm – 3 D with tissue contraction	20	50	70			
Minimal margin <5mm – 3 D without tissue contraction	37	37	74	93% (95% CI: 84,100)	42% (95% CI: 30,54)	62% (95% CI: 52,71)
Minimal margin ≥5mm – 3 D without tissue contraction	3	27	30			
Minimal margin <5mm – 2 D	8	9	17	20% (95% CI: 7,32)	86% (95% CI: 77,94)	61% (95% CI: 51,70)
Minimal margin ≥5mm – 2 D	32	55	87			
Total number of tumors	40	64				

## Data Availability

The data that support the findings of this study are available from the authors on reasonable request.
